# Automated Computer Vision Assessment of Hypomimia in Parkinson Disease: Proof-of-Principle Pilot Study

**DOI:** 10.2196/21037

**Published:** 2021-02-22

**Authors:** Avner Abrami, Steven Gunzler, Camilla Kilbane, Rachel Ostrand, Bryan Ho, Guillermo Cecchi

**Affiliations:** 1 IBM Research – Computational Biology Center Yorktown Heights, NY United States; 2 Parkinson’s and Movement Disorders Center, Neurological Institute University Hospitals Cleveland Medical Center Cleveland, OH United States; 3 Department of Neurology Tufts Medical Center Boston, MA United States

**Keywords:** Parkinson disease, hypomimia, computer vision, telemedicine

## Abstract

**Background:**

Facial expressions require the complex coordination of 43 different facial muscles. Parkinson disease (PD) affects facial musculature leading to “hypomimia” or “masked facies.”

**Objective:**

We aimed to determine whether modern computer vision techniques can be applied to detect masked facies and quantify drug states in PD.

**Methods:**

We trained a convolutional neural network on images extracted from videos of 107 self-identified people with PD, along with 1595 videos of controls, in order to detect PD hypomimia cues. This trained model was applied to clinical interviews of 35 PD patients in their on and off drug motor states, and seven journalist interviews of the actor Alan Alda obtained before and after he was diagnosed with PD.

**Results:**

The algorithm achieved a test set area under the receiver operating characteristic curve of 0.71 on 54 subjects to detect PD hypomimia, compared to a value of 0.75 for trained neurologists using the United Parkinson Disease Rating Scale-III Facial Expression score. Additionally, the model accuracy to classify the on and off drug states in the clinical samples was 63% (22/35), in contrast to an accuracy of 46% (16/35) when using clinical rater scores. Finally, each of Alan Alda’s seven interviews were successfully classified as occurring before (versus after) his diagnosis, with 100% accuracy (7/7).

**Conclusions:**

This proof-of-principle pilot study demonstrated that computer vision holds promise as a valuable tool for PD hypomimia and for monitoring a patient’s motor state in an objective and noninvasive way, particularly given the increasing importance of telemedicine.

## Introduction

Facial expressions are an essential component of interpersonal communication [1]. They depend on our ability to voluntarily and involuntarily contract facial muscles [2], which are innervated by facial nerves. However, neurodegenerative diseases can cause cognitive disorders that affect expressivity [3] and cortical or peripheral nerve traumas [4], and can limit the production of facial expressions and emotion recognition [5,6]. This affects the ability to contract facial muscles by causing hemifacial spasms and can produce involuntary movements (ie, tics) and muscle weakness or stiffness.

Parkinson disease (PD) is a neurodegenerative disease that produces a gradual and generalized loss of motor functions, including the ability to contract facial muscles during spontaneous and voluntary emotional expressions [7], and voluntary nonemotional facial movements [8]. This reduced ability leads to a loss of facial expressiveness that generates a signature “mask-like” appearance of the disease, which is also known as hypomimia. This loss of expressivity is often confounded with depression [2,9], a common symptom in patients with PD. However, even nondepressed PD patients show hypomimia, supporting the hypothesis of a motor control impairment in addition to the effects of depression [2,9]. A hypomimia rating is part of the Unified Parkinson Disease Rating Scale (UPDRS) [10], which is the gold standard clinical assessment tool. When assessing a patient for hypomimia, neurologists rate on a 5-point scale as follows: 0 for normal facial expression, 1 for minimal hypomimia, 2 for slight but abnormal diminution of facial expression, 3 for moderate hypomimia, and 4 for severe or complete loss of facial expression [10].

Disease progression does not seem to produce uniform facial masking across people. Studies of differential deficits in specific muscles [7] and sections of the face have documented asymmetric patterns [11] during posed smiling. However, previous work was based on constrained laboratory tasks where facial expressions were either evoked by sensory stimulations [12] or posed [7,11], limiting the applicability of the results to spontaneous natural expressivity.

In experimental settings, the quantification of masked facies in patients with PD has been traditionally performed with manual scoring [11,13,14]. A method capable of objectively characterizing variations in naturally occurring facial expressions that vary with disease progression would allow the patient state to be continuously evaluated outside of a clinical setting, opening up the possibility of remote or telemedicine-based monitoring. Video analysis has started to demonstrate success in objectively quantifying emotions in psychiatry [15,16] and neurology [17].

Computational methods based on known face components (eyes, mouth/lips, action units, skin, and shape) have been proposed [18-24]. For instance, eye-tracking algorithms [18] have been successful at quantifying the reduction in emotion recognition by patients with PD [19]. These methods involve the analysis of facial movements [22], specific visual features [23], patterns related to a specific emotion such as disgust [12], or facial landmarks [21]. Engineered facial features are nevertheless limited by image quality (distance to camera), pose (nonfrontal looking participant), or visual occlusions (eg, glasses, hands, and hats). These challenges [25] can be overcome by learning features directly from the raw image data using deep convolution networks that are known to be very effective at extracting emotions in healthy participants [26] and to outperform classic feature extraction methods.

Although it is well accepted that PD produces a generalized loss of the ability to produce facial expressions, it is unclear how this deficit evolves with disease progression and what are the effects of dopamine replacement therapy on masked facies [27]. In this work, we describe a methodology to characterize PD hypomimia using deep learning. This procedure can be performed remotely on videos, and thus, it provides a novel noninvasive digital tool for objective assessment of PD hypomimia and the changes associated with an *on*-*off* drug motor state. An automated video-based assessment tool like this one may be valuable for use in telemedicine [28], which has become increasingly utilized in PD especially following the onset of the COVID-19 pandemic. Such a resource would also allow for monitoring of a patient’s motor state at home [29,30] between in-office neurologist or clinical trial visits [31].

## Methods

### Algorithm Development

The neural network model was trained using two data sets of faces, comprising people with PD and controls. The first was the YouTube Faces Database [32] (created by the Computer Science Department of Tel Aviv University), which contains 3425 videos of 1595 people (two-thirds of the subjects are male). The average length of the video clips is 7 seconds. This database constituted the control database for training the Visual Geometry Group neural network [33] in this study. The second training data set was created by performing a search on YouTube using the search terms “Parkinson’s disease” and “interview.” From that search, 107 videos of self-identified PD patients (68 males, middle-aged and older patients) were collected. This latter YouTube set was randomly partitioned into a 75% training set (80/107 videos, 50 males) and 25% test set (27/107 videos, 18 males). By design, this training data set incorporated common image quality challenges (such as varying lightning conditions, poses, and occasional presence of motion blur).

To preprocess the videos, faces were extracted from each frame of each video. Thereafter, each image was converted to grayscale, the intensity was normalized (mean=0.51, standard deviation=0.25), and the image was resized to a standardized 224 × 224 pixels. The neural network was trained using stochastic gradient descent.

**Figure 1:**
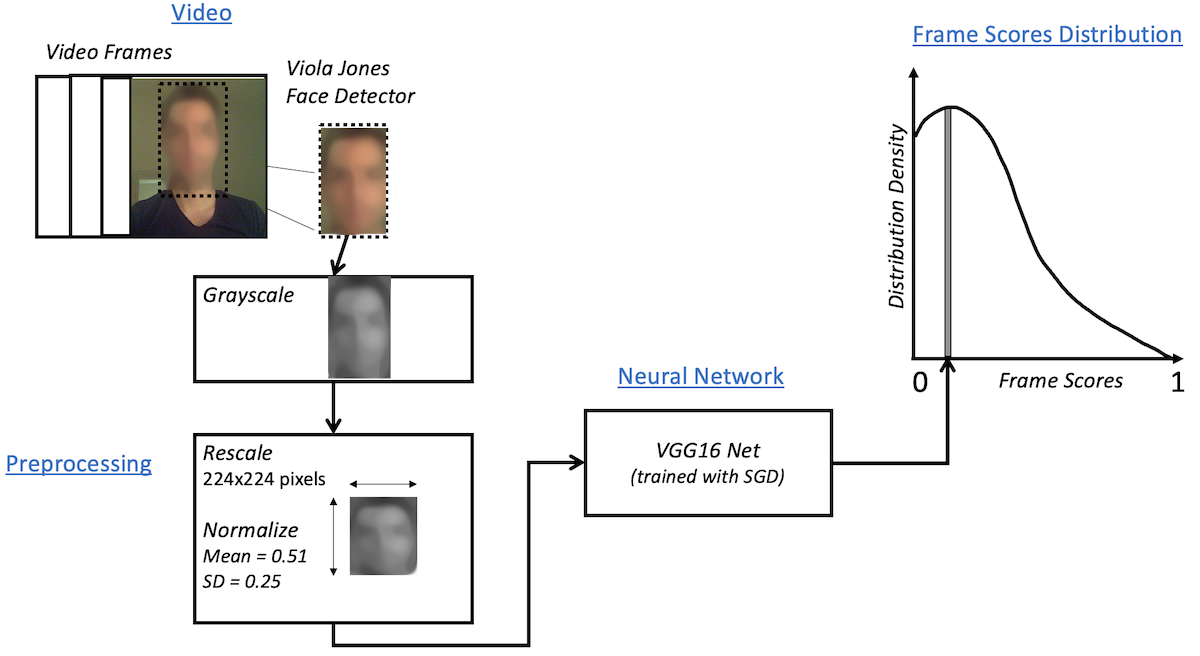
The preprocessing pipeline for the input videos. Faces are extracted, greyscaled, and normalized. Then, each frame in the video is assigned a probabilistic classification assignment from 0 to 1 representing the degree of hypomimia. Thus, each video is represented by a probability distribution of frame scores. SGD: stochastic gradient descent.

After training, for each new video in the test set, the algorithm assigned each frame a score between 0 and 1, based on the degree of hypomimia that was detected by the algorithm in that frame. The scores of all of the frames of a video together formed a density distribution for that video (Figure 1), which demonstrated the proportion of frames that are assigned each likelihood of hypomimia. It is important to note that not all frames are indicative of the disease state, as a patient with PD may well have some frames where he/she does not exhibit hypomimia. Thus, the probability distribution for each video (and thus each subject) had a different shape proportional to the underlying hypomimia severity. We hypothesize that a PD video will have more frames with a higher hypomimia score than will a control video. Similarly, we hypothesize that a patient with PD in the *off* drug state will have more frames with a higher hypomimia score than that same subject in the *on* drug state.

**Figure 2:**
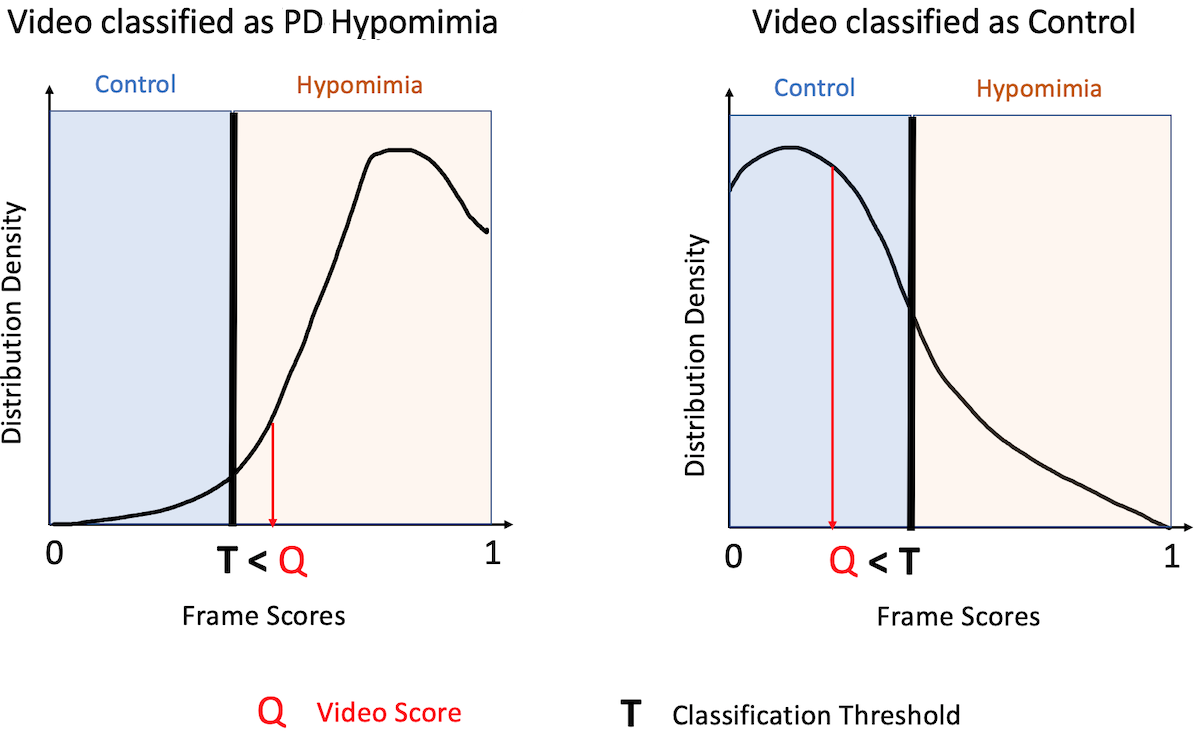
Video scoring. To classify a video, a probability distribution is created for all of a video’s frames, and the fifth percentile of the distribution is defined as Q. A video that has a Q value above T (ie, closer to 1 or more evidence of hypomimia) is categorized as PD hypomimia; a video with a Q value below T (ie, closer to 0 or less evidence of hypomimia) is categorized as Control. PD: Parkinson disease.

In order to classify each video, we needed to characterize this density distribution for each video as a single number. To do so, we took the fifth quantile (Q) of that video’s frame score density distribution (other quantiles can be used without loss of generality as discussed in the Results section). A video that exhibits low hypomimia should have a positively skewed distribution, as the bulk of the probability mass will be closer to 0, and therefore, will have a lower value of Q. In contrast, a video that exhibits high hypomimia should have a negatively skewed distribution, with the bulk of the probability mass closer to 1, and therefore, will have a higher value of Q. Thus, the value of Q can be used to characterize how strongly hypomimia is detected in a given video, by representing how far along the 0 to 1 continuum is required to achieve 5% of the video’s frames. Using this metric, we hypothesize that control videos will have a relatively lower Q (more frames concentrated toward 0) and PD videos will have a relatively higher Q (more frames concentrated toward 1; Figure 2).

Finally, a classification threshold T was selected. Any video that had a Q value lower than the threshold T was classified as not PD (ie, 0). Any video that had a Q value higher than the threshold T was classified as PD (ie, 1). T was selected such that it maximized classification accuracy in the testing set and was validated using the separate held-out validation set consisting of the Alan Alda videos.

### Algorithm Testing

The difference in video scores between the PD and control groups was tested on a set of 54 videos (middle-aged and older patients, 37 males). Of these, half (n=27) featured people with self-identified PD, and the other half (n=27) featured people without PD (controls). The control videos were selected to include people who self-reported having other neurological or psychiatric disorders, with the following breakdown: 18 healthy people, four people with depression, one person with posttraumatic stress disorder, one person with traumatic brain injury, one person with bipolar disorder, one person with schizophrenia, and one person with chronic back pain. For the videos that were categorized as PD or other disorders, identification was performed based on the uploader’s self-report (ie, the title of the video), not a clinical evaluation. However, many of the videos were created by disease associations, clinicians, academics, documentaries, or celebrities who publicly revealed their diagnoses, providing some degree of confidence of the reliability of the self-report.

### Algorithm Validation

#### Hypomimia and Drug State

The Tufts Clinical data set consists of 35 participants (mean age 68 years, SD 8 years; 23 males and 12 females; mean total UPDRS-III score 25, SD 13) with idiopathic PD. The protocol was run at Tufts Medical Center in Boston, Massachusetts and was approved by the Tufts Health Sciences Campus Institutional Review Board (IRB #12371) (the complete study design [34] and related analyses conducted on the data set [35-37] have been reported previously). Patients were video recorded by means of a Microsoft Kinect camera (Microsoft Corp) at 30 frames per second.

Only 33 patients participated in a clinical interview in both their *on* and *off* drug states, with a mean of 3639 frames per video (approximately 2 minutes), which is similar to the length of the videos used in the training data set.

All 35 patients performed the UPDRS-III scripted tasks (including pronation-supination, finger tapping, and walking) and simulated activities of daily living [34] (including book carrying, bottle shaking, coat buttoning, cursive writing, and zipping) during their clinical visit, with a mean of 50,987 frames per video (approximately 28 minutes).

PD medication state (*on* or *off*) was self-reported by the participant at the start of each session. Medication dosage and timing was determined by the participant’s typical daily dosage of levodopa (L-DOPA) therapy. Participants refrained from taking additional dosages in order to follow this experimental protocol. Participants were randomly assigned to an order condition (either completing the protocol in *on* first or *off* first). All participants arrived at the clinic in the *off* state. If they were assigned to the *off* first condition, they completed the experimental protocol when they arrived in the clinic (*off* state). Thereafter, they took their scheduled L-DOPA dose and waited until the medication’s effects began. They were evaluated by the neurologist administering the UPDRS every 30 minutes until they self-reported being in the *on* medication state or 1.5 hours after the dose (whichever came earlier). Once this occurred, they completed the experimental protocol a second time (*on* state). In contrast, if the participant was assigned to the *on* first condition, they took their scheduled L-DOPA dose once they arrived at the clinic, waited until its effects began (as described above), and then completed the experimental protocol for the first time (*on* state). These participants then left the clinic and came back for a second scheduled session on a different day to perform the experimental protocol in the *off* state.

The UPDRS-III Facial Expression item, which rates the impairment of facial expressions, was used as the reference outcome variable in the present analyses. We characterized a strictly positive UPDRS-III Facial Expression score (ie, a rating greater than 0) as a positive PD classification by the examining neurologist. Additionally, we characterized a strictly positive difference of the UPDRS-III Facial Expression score between *off* and *on* (*off* minus *on*) as corresponding to a positive drug state classification by the neurologist.

Participants should have less dysfunction (and a lower UPDRS score) when they are in the *on* medication state than when they are in the *off* medication state. The present work investigates the effectiveness of the proposed computer vision algorithm to detect hypomimia in these patients, as well as quantify their medication state (ie, *on*/*off*) by detecting hypomimia. In that respect, if the algorithm is predictive of a patient’s drug state, the model should predict a lower score for the *on* state as levodopa contributes to lowering PD symptoms by increasing the availability of dopamine to the brain. This hypothesis was tested by computing the change in score between the *off* and *on* medication states.

#### Longitudinal Severities of Masked Facies

The longitudinal data set consisted of seven videos of public appearances of Alan Alda from 1974 to 2019 (age 38-83 years), in which he was engaged in public speaking. Alan Alda is an actor, director, and screenwriter who was diagnosed with PD in 2014. This data set consists of four videos before diagnosis and three videos after diagnosis, and is used to evaluate the present algorithm’s ability to quantify hypomimia. In this data set, a mean of 9642 frames per video (5.3 minutes) was extracted and analyzed by the algorithm. In these interviews, Mr Alda is recorded in diverse poses and lightning conditions, making the longitudinal data set qualitatively similar to the training data set and Tufts Clinical data set.

## Results

### Hypomimia Detection (Test Set)

**Figure 3:**
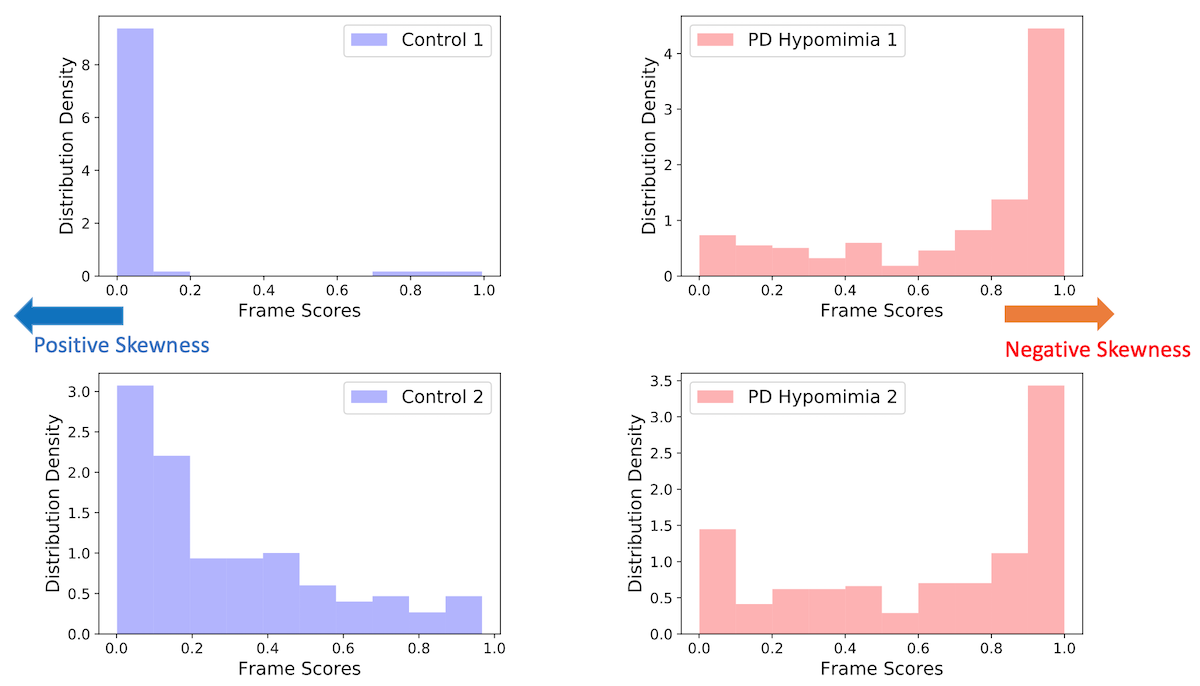
Example test set of PD and control distributions for four videos from the test data set. Two control videos (in blue) and two PD videos (in red). The distributions of the PD videos have higher weight on score values closer to 1 (negatively skewed) compared to the control videos, thereby demonstrating greater incidence of hypomimia. PD: Parkinson disease.

As expected for the PD videos, a greater proportion of frames were classified as “PD hypomimia” than were for the control videos. The skewness of the PD subject video distributions was significantly smaller than that of the control videos (one-tailed Mann-Whitney *U*=212, *P*=.004), demonstrating that there was more weight to the right side of the distribution (hypomimia scores closer to 1) for PD videos than for control videos (Figure 3).

**Figure 4:**
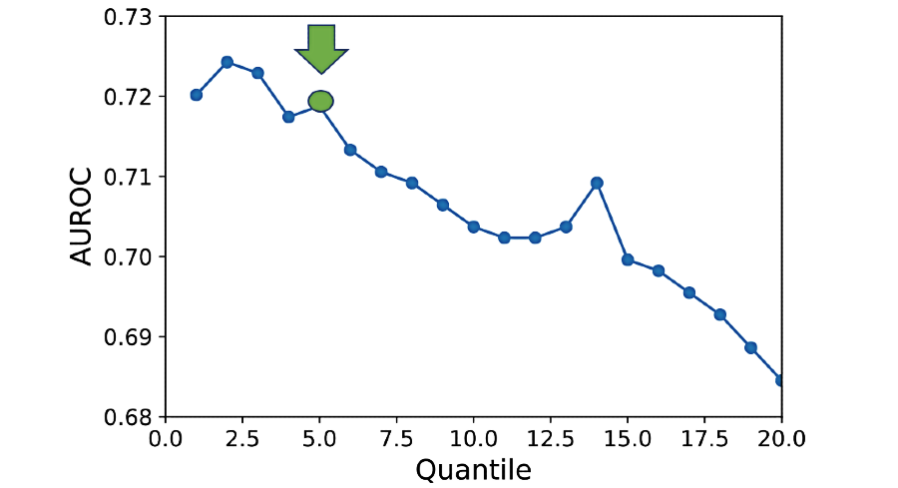
Test set AUROC (PD vs Control) as a function of the chosen video distribution quantile to use as the “Q” threshold. A wide range of quantiles achieves AUROC > 0.7 (15th quantile and below). The fifth quantile, selected as our video score threshold, is shown in green. AUROC: area under the receiver operating characteristic curve; PD: Parkinson disease.

We experimentally quantify this difference in skewness by selecting the fifth quantile Q, which becomes the video score. The greater the incidence of hypomimia in the frames of a given video, the higher the quantile Q. Figure 4 shows that a wide range of quantiles would provide satisfying results on the test set (all quantiles below 15 achieve an area under the receiver operating characteristic curve [AUROC] >0.7). We chose the fifth quantile without loss of generality and applied this choice to the validation data sets only. A classification threshold (T=0.0003) was selected to maximize classification accuracy (70% accuracy or 38/54 videos correctly classified) in the test set. This threshold was determined on the basis of performance on these test set videos and then evaluated on the separate held-out validation data sets (Alan Alda and Tufts Clinical) to characterize hypomimia cues.

To provide a baseline accuracy measure, two professional neurologists rated each video in the test data set on the UPDRS-III Facial Expression score (score between 0 and 4). The neurologists performed the evaluation on the video, not an in-person clinical examination, and were told just to focus on the Facial Expression score and attempt to avoid being influenced by other cues present in the subject’s behavior, to the extent possible. Using this scoring system, one neurologist’s ratings produced an AUROC of 0.64 and the other neurologist’s ratings produced an AUROC of 0.79. Averaging both neurologists’ UPDRS-III Facial Expression scores produced an AUROC of 0.75. These scores were taken as an approximation of baseline classification accuracy that could be achieved using expert human raters. It is important to note, however, that this accuracy is an approximation and a true in-person clinical rating would incorporate substantially more information than just the UPDRS-III Facial Expression score.

### Hypomimia Changes Associated With the Drug State in the Tufts Data Set (Validation Set)

Finally, we assessed the performance of our algorithm on the held-out validation sets, after being trained on the training set and accuracy maximized on the testing set. The first validation set was the Tufts Clinical data set used to assess hypomimia changes associated with the drug state. For each patient, we extracted and analyzed all frames in the video. Our goal was to quantify PD hypomimia for each patient’s visit and see if *on* and *off* motor states had an impact on hypomimia as captured by the neurologist’s UPDRS-III Facial Expression score and our algorithm.

To test if the algorithm was able to correctly classify PD hypomimia, we computed the score of each clinical interview video to see if it exceeded the decision threshold T. If the score was above the threshold, the video was categorized as PD. The algorithm detected PD in 76% (25/33) of the *off* drug sessions (in comparison, the neurologist gave a UPDRS-III Facial Expression score higher than 0 for 88% [29/33] of these sessions) and in 67% (22/33) of the *on* drug sessions (in comparison, it was 70% [23/33] for the neurologist). This reduction in facial masking detection between the *off* and *on* drug sessions for both the algorithm and the neurologist can be attributed to drug efficacy in reducing PD symptoms.

To quantitatively evaluate the difference between the *off* and *on* states, we computed the difference between the *off* and *on* video scores for each participant. The *off* score generated by the algorithm was strictly greater than the *on* score in 63% (22/35) of the participants during the clinical visit. In comparison, the neurologist ratings of the UPDRS-III Facial Expression score were strictly greater in the *off* state than in the *on* state for only 46% (16/35) of participants. However, it is worth noting that the UPDRS-III Facial Expression score is integer based and does not allow clinicians to assess changes in facial expression that are more granular than these integer ratings. The clinical interview accuracies of the UPDRS-III Facial Expression score and algorithm were 45% (15/33) and 55% (18/33), respectively.

**Figure 5:**
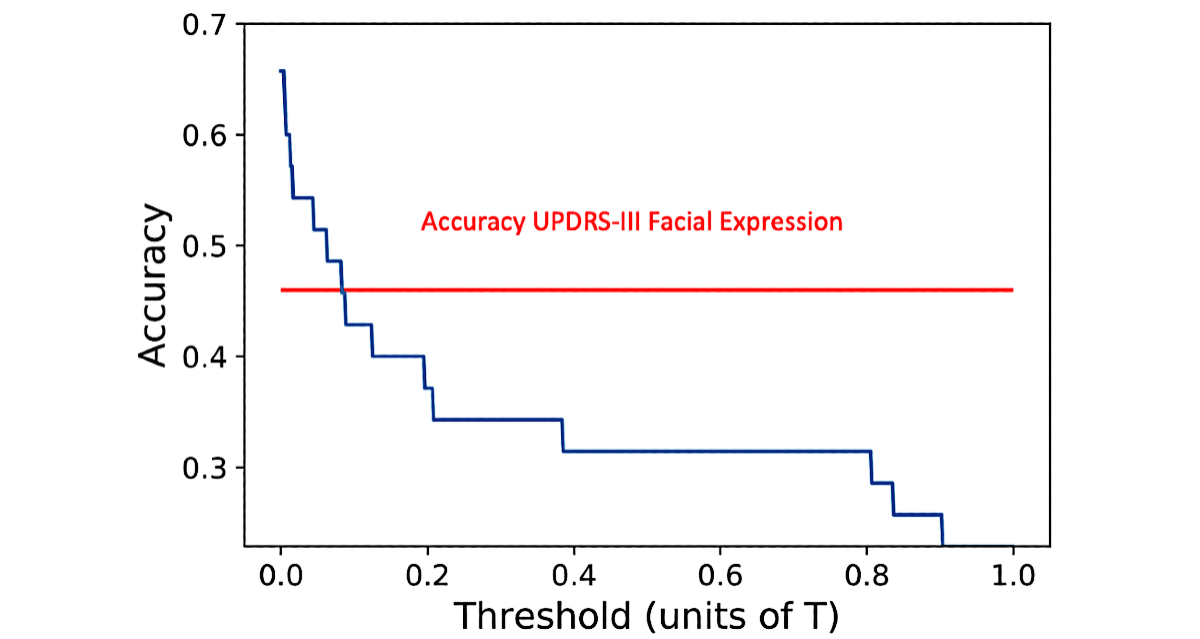
On-off sensitivity of the Tufts data set. On-off classification for a clinical visit is displayed as a function of threshold. On-off differences are far more subtle than PD versus Control differences (on which the model was originally trained). The red line shows the percentage of participants for which the neurologists rated the UPDRS-III Facial Expression score higher in the off state than in the on state. PD: Parkinson disease; UPDRS: United Parkinson Disease Rating Scale.

To quantify the sensitivity of our analysis, we provided a plot highlighting the differences in detection as given by different thresholds (the x-axis is scaled by T). Thresholds to separate the *on* state from the *off* state effectively were smaller than T, which appeared reasonable as we expected the difference between the *on* and *off* states to be more subtle than the difference between the PD and control groups. More precisely, a threshold of 0.1 T provided an accuracy comparable to that of the neurologist (46% accuracy), and a threshold of 0.01 T led to 60% accuracy (Figure 5).

### Longitudinal Severities of Masked Facies

**Figure 6:**
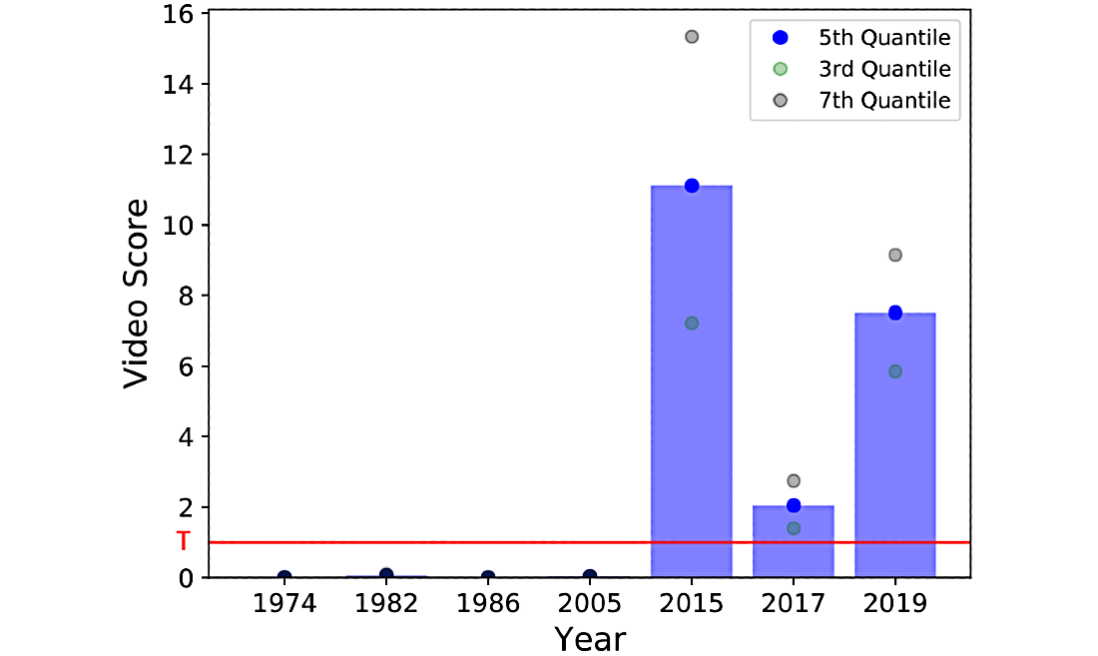
Validation with Alan Alda interviews. All videos after Mr Alda’s PD diagnosis are above the threshold T, whereas videos before his PD diagnosis are below T (horizontal red line), indicating that the algorithm is sensitive to PD hypomimia symptomatology. Dots show the confidence interval (third quantile and seventh quantile of the video density distribution). PD: Parkinson disease.

We sought to retrospectively validate the algorithm’s ability to characterize PD symptomology in an individual longitudinally. The algorithm was applied to seven interview videos featuring Alan Alda (officially diagnosed with PD in 2014) from 1974 to 2019. There was an increase in the algorithm’s PD classification before PD diagnosis to after diagnosis. Indeed, all videos before PD diagnosis were below the optimal threshold T for positive classification, and all videos after diagnosis were well above the threshold, highlighting the fact that the algorithm was able to capture hypomimia cues. Finally, we included a confidence interval (as given by the third and seventh video distribution quantiles) associated with the video scores (Figure 6).

## Discussion

### Principal Results

In this proof-of-principle pilot study, we used deep learning to detect PD hypomimia from videos of people with and without PD. Our method was also able to detect the effect of dopamine replacement medication in participants during their clinical visit and to analyze the progression of symptoms in the actor Alan Alda before and after his diagnosis of PD.

### Comparison With Prior Work and an Alternative Approach

A well-established method to identify facial expressions was proposed by Ekman and Friesen [38], which describes visual facial movements related to the muscles involved in the production of emotions. Known as the Facial Action Coding System (FACS), this method uses localized image information and has been previously applied to study parkinsonism [12,39]. However, the studies did not provide information on medication state or longitudinal changes of facial expressions in PD patients.

An alternative approach to investigate the progression of PD as a function of the ability to move specific facial muscles is to use electromyography [7]. This approach may be considered less prone to artifacts of head movement, complexion, and facial bone structure, but the use of electromyography at a participant’s home is technically challenging and impractical.

### Limitations

There are noteworthy limitations to our work. The training data set is limited, as in particular, it did not include people in the full range of relevant ages affected by PD (early onset PD patients were not represented), which constrains generalizability. The effect of dopaminergic medication was not taken into consideration when training the model, as all videos in the training data sets were classified as either PD or control, with no consideration of *on* versus *off* medication state. There is uncertainty linked to the video labels in the training data set, as it relied on the uploader’s self-report (ie, the title of the video), not a clinical evaluation. Consequently, it is important to validate the present algorithm with clinically verified participants with and without PD, as was performed in this work using clinically validated participants in *on* and *off* drug states. Moreover, while gender and, to a lesser extent, age can be ascertained with some degree of certainty for subjects in the training data set, additional demographic information is highly limited, making generalization to larger test sets potentially susceptible to demographic or other biases in the training sets. This observation, coupled with the fact that the longitudinal study is limited as it was applied on only seven videos of one person, implies that more extensively curated training data sets as well as larger testing data sets will be required to validate the robustness of our method.

### Conclusions

Our algorithm may serve as a nonclinical marker for PD hypomimia and *on* and *off* motor states. Unlike the lengthy physical examination techniques required for the clinical assessment of PD, which require in-person or video examinations that must be rated by a trained clinician, the present automated technique is capable of rating videos of a patient’s face. This technique has the potential to improve the ability to continuously monitor the *on* and *off* states, even in the patient’s home. This can thereby serve as a serial data point for use in at-home monitoring for PD or at-home assessment in a PD clinical research study, with little patient burden and minimal technological requirements.

With a shift toward a greater role of telemedicine, an automated assessment of hypomimia could serve as a screening tool for parkinsonism and as a nonobtrusive objective score to assess *on* and *off* states. Further study will be needed to assess the value of this automated assessment in various clinical settings. The proposed model was tested on PD hypomimia, but in theory, it could be applied to other neurological conditions that produce other face signatures.

## Abbreviations

AUROC: area under the receiver operating characteristic curve
L-DOPA: levodopa
PD: Parkinson disease
UPDRS: United Parkinson Disease Rating Scale
